# A Comprehensive Analysis of Recent Deep and Federated-Learning-Based Methodologies for Brain Tumor Diagnosis

**DOI:** 10.3390/jpm12020275

**Published:** 2022-02-13

**Authors:** Ahmad Naeem, Tayyaba Anees, Rizwan Ali Naqvi, Woong-Kee Loh

**Affiliations:** 1 Department of Computer Science, University of Management and Technology, Lahore 54000, Pakistan; f2019288007@umt.edu.pk; 2 Department of Software Engineering, University of Management and Technology, Lahore 54000, Pakistan; tayyaba.anees@umt.edu.pk; 3 Department of Unmanned Vehicle Engineering, Sejong University, Seoul 05006, Korea; 4 School of Computing, Gachon University, Seongnam 13120, Korea

**Keywords:** brain tumor, deep learning, federated learning, tumor diagnosis, tumor detection, magnetic resonance imaging, health care

## Abstract

Brain tumors are a deadly disease with a high mortality rate. Early diagnosis of brain tumors improves treatment, which results in a better survival rate for patients. Artificial intelligence (AI) has recently emerged as an assistive technology for the early diagnosis of tumors, and AI is the primary focus of researchers in the diagnosis of brain tumors. This study provides an overview of recent research on the diagnosis of brain tumors using federated and deep learning methods. The primary objective is to explore the performance of deep and federated learning methods and evaluate their accuracy in the diagnosis process. A systematic literature review is provided, discussing the open issues and challenges, which are likely to guide future researchers working in the field of brain tumor diagnosis.

## 1. Introduction

Cancer is a significant cause of death worldwide, as revealed in research done by the World Health Organization (WHO) [1]. It is predicted that in the coming years, the rate of cancer in people will double [2]. Early diagnosis and treatment of cancer can reduce the risk of mortality. Thus, in the field of neuroscience, the main interest of researchers is to develop a system for the early detection of brain cancer [3]. Brain tumors are the deadliest cancer, with a high mortality rate compared to the number of new cases per annum. More than 250,000 new cases of brain and nervous system cancers have occurred worldwide [2]. In brain tumors, tissues grow abnormally, and these tissues do not perform any brain function except for the uncontrolled multiplication of cells. Brian tumors cause abnormal neurological disorders, which increase the pressure and size of the brain. They also cause brain swelling. In developing countries, the number of people who die from brain tumors has increased by 300 percent according to the National Brain Tumor Foundation (NBTF) [4,5]. The National Brain Tumor Society (USA) reports released in 2020 indicate that 700,000 people in the United States have been living with a brain tumor. Brain tumor cases have risen steadily over the last 30 years, similar to other cases of cancer. If a brain tumor is diagnosed in the early stages, minor surgery, chemotherapy, and radiotherapy can increase the chances of recovery [6].

The primary purpose of the computerized diagnosis of brain tumors is to collect clinical knowledge about the presence, location, and type of tumor. Information from clinical imaging is utilized for the correct diagnosis and treatment of cancer. Automated diagnosis of brain tumors includes multiple strategies that can be hierarchically arranged. Different techniques for planning, labeling, selecting, and explaining data are needed at each stage of the hierarchy. Despite a reasonable amount of work done in this field, however, clinicians still depend on manual tumor projections. This is probably due to a lack of communication between clinicians and researchers. There is a need for an efficient automated system for the early detection of brain tumors to help decrease the mortality rate [7]. The chances of patient survival increase if the tumor is diagnosed at an early stage. Proper diagnosis of the location, size, shape, and type of tumor is of particular importance. For this purpose, brain-imaging techniques such as positron emission tomography (PET), computed tomography (CT), magnetic resonance spectroscopy (MRS), and magnetic resonance imaging (MRI) have been widely used. MRI and CT scans are popular techniques because of their wide availability. MRI generates images of human tissues by utilizing a strong magnetic field with radio frequency signals, providing detailed information on the anatomy of human tissues, whereas CT scans use X-rays to build interior images of the body from different angles [8,9]. The diagnosis of brain tumors is based on three key steps: tumor sensing, segmentation, and classification. Brain tumor segmentation techniques are used to separate different tumor tissues from MRI images, and classification methods are applied to these tissues. Abnormal images are classified as malignant or benign with the help of these techniques [10]. Over the past few decades, several studies, providing a significant amount of research on brain tumor diagnosis, have been conducted. These studies present methods for the segmentation and classification of tumors. 

AI-based methods are used for brain tumor detection because of their outstanding results [11]. AI technologies have been implemented in the field of e-healthcare systems, with numerous advancements in medical science. These techniques help domain experts provide better health care to patients [10]. Progress in deep learning, which is a combination of AI and machine learning, has contributed to many state-of-the-art brain tumor identification solutions, allowing early cancer discovery, whereby preemptive measures can be taken to save lives [12]. However, the results of deep learning are less accurate, as the datasets for training and testing are smaller. To overcome this issue, federated learning is used to train the shared global model using data from several institutions without compromising data privacy [13].

Few studies have reviewed deep and federated learning. Moreover, these studies have limitations. Nalepa et al. [11] reviewed current techniques for data augmentation using MRI images. The main focus of this review was to investigate papers submitted to the multimodal brain tumor segmentation challenge BraTS 2018. The study focused on the practical aspects of the proposed algorithm, concentrating on the BraTS dataset. Information on unpublished, combined datasets and images collected from the internet were not included in the study. Another systematic literature review was performed by Abd-Ellah et al. [14] on brain tumor detection, segmentation, and classification using machine and deep learning, but they did not provide an overview of recent deep learning methods and the BraTS 2018 dataset. Despite the general success of AI in diagnosis, it is still challenging to build an effective model with minimal datasets, at particular sites. To address this issue, federated learning is utilized to train the global model across individual sites. Federated learning is the process of integrating training results from numerous sites to generate a global model without directly exchanging information. This work focuses entirely on federated learning and its challenges [15]. 

This study examines the essential existing diagnostic methods for brain tumors and focuses on fundamental deep learning and federated learning methods using MRI images for brain tumor diagnosis. In addition, it provides a systematic analysis of the federated and deep learning literature on brain tumor detection and segmentation, while mainly focusing on classification. In recent years, much work has been performed on the automated diagnosis of brain tumors using deep learning, whereas only a few studies have been conducted on federated learning. Thus, it is critical to compile, review, identify, and encapsulate state-of-the-art work. This paper describes the proposed taxonomy for brain tumor detection by analyzing existing research based on deep learning and federated learning. Furthermore, various deep and federated learning methods, which use benchmark datasets to detect and classify brain tumors, are discussed. This paper highlights the open issues and challenges that exist in the field of brain tumor detection using deep and federated learning, analyzing various datasets obtained from public and non-public repositories. Future research areas are identified and the main shortcomings of existing methods are further categorized.

This study comprises seven sections. The introduction and objectives of this study are presented in Section 1. The research procedure is discussed in Section 2. The findings of each selected paper are discussed in Section 3. The taxonomy and model are proposed by analyzing the selected papers in Section 4. Open issues and challenges are discussed in Section 5. The principal findings of this study are presented in Section 6. Finally, Section 7 concludes the paper.

## 2. Research Method

**Figure 1:**
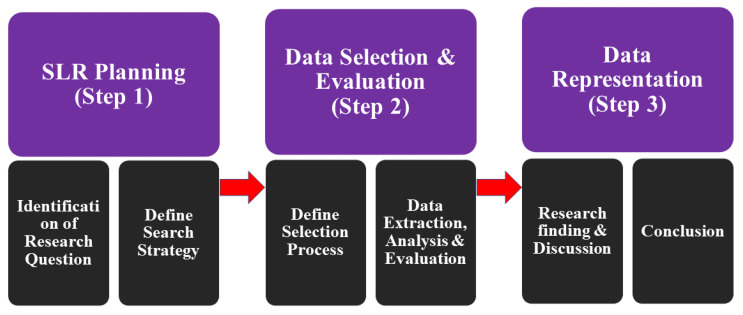
Key Steps for SLR.

The purpose of this systematic literature review (SLR) is to categorize the state-of-the-art methods for the diagnosis of brain tumors. In Figure 1, the key steps are outlined for the systematic review.

### 2.1. Objectives of Research

The primary goals of this study are:

- Focusing on the latest research on brain tumor diagnosis using deep and federated learning.
- Identifying current research trends, open issues, and challenges for brain tumor diagnosis.
- Investigating current brain tumor diagnosis approaches based on similarities and discrepancies.
- Proposing a taxonomy for brain tumor detection subsequent to an analysis of effective methods.

### 2.2. Research Questions

The research questions were constructed based on research problems in a particular domain. After identifying the research questions, the specific area of the research problem was analyzed. Kitchenham et al. [16] proposed a methodology to identify answers to defined questions through published literature. The primary focus of this study is to summarize current state-of-the-art brain tumor diagnosis methods using deep learning. To determine the significance of the study, research questions have been formulated in Table 1. 

### 2.3. Search Strategy

The search strategy for the extraction of appropriate information from focused areas and the elimination of unrelated studies constitute the foundation for well-organized research [17]. In this systematic study, articles that developed deep-learning-based methods for brain tumor diagnosis have been shortlisted. For this purpose, Elsevier, ACM, IEEE, Springer, MDPI, Wiley, Miccai, and Medline were searched from 1 January 2017, to 20 December 2021, to retrieve relevant articles. Other digital libraries were also explored but were not included due to accessibility constraints. In Table 2, the search strings for repositories are listed [18]. The search string uses primary, secondary, and added keywords. Table 2 shows the keywords for the search string.

### 2.4. Study Screening Criteria

In this article, only those research papers that are aligned with the objectives of this study were included. All other searched articles that were not relevant to the research questions were omitted. Hence, an assessment was carried out to check the relevance of these articles. For the screening of relevant papers, the defined search process by Dybå et al. [19] was adopted. Research papers were included based on the search strings [20]. Articles were excluded based on the following criteria [21]:

- Research articles not based on binary disease classification.
- Research articles diagnosing brain tumors without medical images.
- Research articles not identifying data sources or employing ambiguous methods of data collection.
- Research articles based on non-human samples.

### 2.5. Study Selection Process

**Figure 2:**
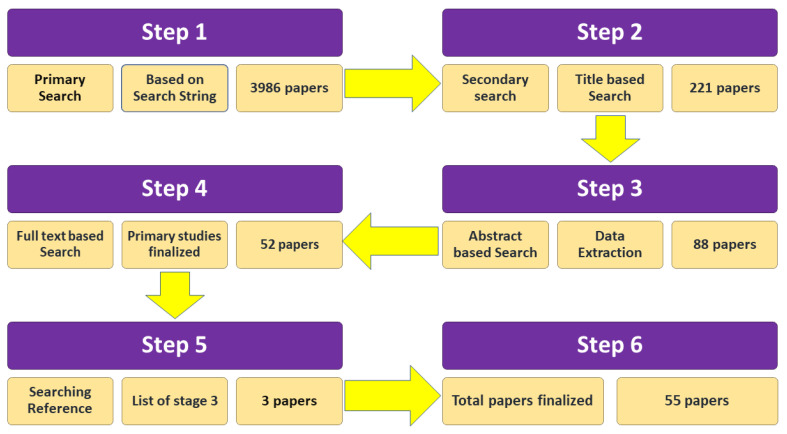
Selection and screening process.

The most relevant research aligning with the objectives of this study is included [22]. In the first phase, 3986 studies were identified using the search strings. In the second phase, irrelevant and duplicate papers were omitted manually by analyzing the titles. At this stage, 221 articles were classified as appropriate. The abstracts of the research papers were reviewed in the third stage. After that stage, 88 papers remained on the list. The next stage was to provide a full-text-based review. At this stage, 52 papers were considered for the analysis. Moreover, snowball tracking was carried out by searching the references of selected studies to ensure that no important research was missing. The references of each selected paper were analyzed thoroughly, and three more articles were thus included in the list of selected papers, resulting in a total of 55 primary studies. Figure 2 displays the total number of selected research papers.

## 3. Data Analysis and Results

A brief assessment of each study is provided in this section. The strengths and weaknesses of each study are discussed. A summary of each study is provided in tabular format as well. 

### 3.1. Search Results

**Figure 3:**
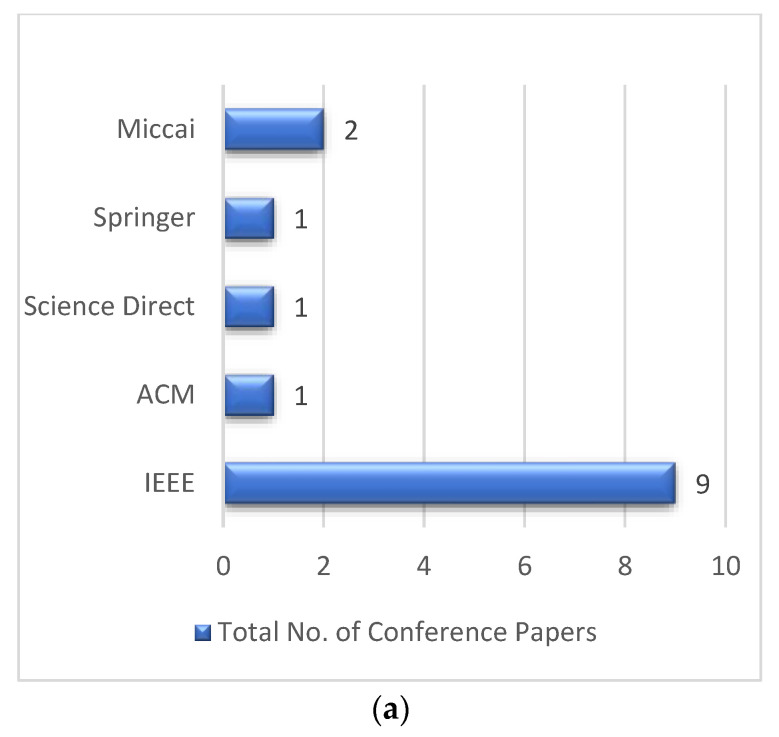

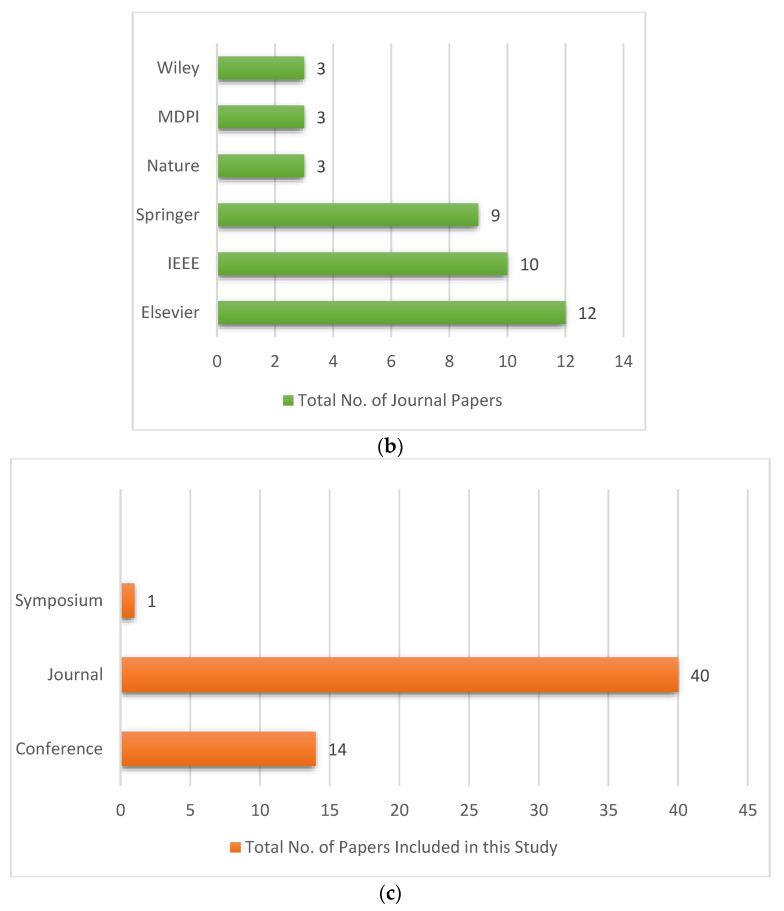
Distribution of (**a**) selected conference papers and (**b**) selected journal papers; (**c**) distribution of total selected papers.

The 55 selected papers were collected from different publication channels, which include journals, conferences, and symposiums. Figure 3c showed the total number of papers selected in this study. The selected conference papers were collected from different repositories. Bar graphs were used to represent different repositories such as IEEE, ACM, Springer, Miccai, and Science Direct, as shown in Figure 3a. The distribution of selected journal papers from different repositories are shown in Figure 3b.

### 3.2. Discussion and Evaluation of Research Questions 

The findings of the research questions are described in this section. Each selected study was used to answer individual research questions. Most of the detailed work in this area has depended on novel techniques used for brain tumor detection, segmentation, and classification using deep learning and federated learning [8]. After a detailed review of the selected papers, conclusions were drawn. 

### 3.3. Analysis of RQ1: What Are the Best Available Methods for the Detection of Brain Tumors?

In this study, every question was analyzed according to the information extracted from the selected studies. In the field of deep learning and federated learning, there are numerous research techniques for tumor diagnosis. In this section, state-of-the-art techniques are examined. Table 3 provides a summary of the different brain tumor diagnosis techniques. 

#### 3.3.1. Pretrained Classifiers

To overcome the issues of convolutional neural networks, a new deep architecture named CapsNets was proposed by Parnian et al. [23] which is highly vulnerable to the diverse backgrounds in images and accesses the surrounding tissues of the brain tumor. This technique achieves promising results compared to traditional methods. The Figshare dataset, which contains 3064 images collected from 233 patients, was used. Moreover, the reliability of CapsNet increases if more datasets are used. Dong et al. [24] applied U-Net with a CNN to automatically and efficiently segment brain tumors. For the evaluation of this method, the brain tumor segmentation (BraTS) 2015 dataset was used, including both low-grade and high-grade tumor cases. This study uses only one dataset to validate the strength of Unet, which is the major drawback. Laukamp et al. [25] proposed a deep-learning-based method to check the reliability of the detection and segmentation of brain tumors using multiparametric images from various institutions. The proposed method was not validated on publicly available datasets. Kotowski et al. [26] proposed a method that implements U-Net for the detection and segmentation of brain tumors. The BraTS 2019 dataset was used to validate the performance of the proposed method. The main shortcoming of these pre-trained methods is that the efficiency of the proposed methods was only measured on a single dataset. Multiple datasets must be used to measure the effectiveness of the proposed method. 

#### 3.3.2. Handcrafted Classifiers

Cui et al. [27] classified brain tumors by cascading them into two stages. In the first step, CNN was utilized for learning, and in the second stage, the trained set was transferred to a support vector machine (SVM)-based classifier for the segmentation of brain tumors. This technique uses an image dataset collected from 30 patients. The major drawback of this study is the utilization of fewer numbers of patient data. More data from patients can enhance the effectiveness of the proposed method. Pathak et al. [28] also utilized CNN to classify brain tumors, and for the segmentation of the tumor, the watershed technique was used for effective image retrieval. A total of 330 images were used to verify the efficiency of the algorithm. This study did not use the publicly available datasets to check the strength of the proposed method. Zhao et al. [29] proposed a CNN-based model called hybrid two-path convolution, which utilizes a different path to collect fine and coarse features. Fine-tuning was applied to achieve the best results. The BraTS 2017 dataset, which contains 285 images, was used for experimentation. Only one publicly available dataset is used for experimentation in this study, and more public datasets can enhance the reliability of the proposed system. CNN was further incorporated by Muthu et al. [30], who employed pre-processed maps to identify the MRI brain image in the curvelet domain. The curvelet extracts features with good resolution and directionality. This study uses only 100 Dicom images, which were collected from different publicly available datasets. At least one public dataset must be used to validate the strength of the proposed system. Soleymanifard et al. [31] utilized the CNN classification technique and active contours for the segmentation of brain tumors. This technique recognizes the boundaries of the tumor faster and focuses on the immediate area of the tumor. They used the BraTS 2015 dataset to validate the results. This study only focuses on BraTS 2015 dataset. More publically available datasets must be used. Özyurt et al. [32] proposed a method that uses a CNN with extreme learning and fuzzy c-means with super-resolution, whereby the brain tumor was segmented using fuzzy c-means for the detection of the pre-trained tumor. SqueezNet was utilized, and the cancer genome atlas glioblastoma multiform (TCGA-GBM) database was used for the images. This study used 150 malignant and 150 benign images in the Dicom format. More images can enhance the reliability of the proposed method. Another novel idea proposed by Sharif et al. [33] achieved precise segmentation with the aid of triangular fuzzy median filters using extreme learning machine (ELM) for classification. The efficiency of the model was tested on the BraTS 2012–2015 datasets, which validated the strength of the proposed method. Thillaikkarasi et al. [34] provided a method in which the tumor was automatically and efficiently segmented using a novel deep learning algorithm with multiclass-SVM (M-SVM). This work involved many steps, including preprocessing, extraction of features, classification of images, and division of the brain tumor; a total of 40 MRI images were used to test this method. A few MR images were used in this study. The proposed system must be tested on publically available datasets. Myronenko et al. [35] proposed a deep-learning-method-based 3D semantic segmentation with conventional encoder-decoder architecture. The BraTS 2019 dataset was used to validate the proposed method. More datasets must be included to check the efficiency of the proposed method.

#### 3.3.3. Ensemble Classifiers

Madhupriy et al. [36] used a deep neural network and probabilistic neural network for the detection of brain tumors. This method works efficiently on low-level and high-level tumor grades because they can exist in any location within the brain, in different shapes and sizes. This system efficiently segments abnormal brain tissues. The BraTS 2013 dataset was used to determine the efficiency of the system. An innovative idea was proposed by Vijh et al. [37], who achieved an optimal threshold value by using the clustering-based image thresholding method OTSU with adaptive swarm optimization. For noise removal, they use an anisotropic diffusion filter, whereas classification is performed by a CNN. The reliability of the method was verified on 101 MR images. The performance of ensemble classifiers works best on large datasets, more data validates the accuracy of the classifier. 

#### 3.3.4. Federated Learning

Sheller et al. first proposed federated learning (FL) to improve the training process. For this purpose, multiple organizations collaborated to protect the privacy of patient data. The performance of federated semantic segmentation was demonstrated using a deep learning model [38]. Li et al. applied federated learning based on a deep neural network (DNN) for the segmentation of brain tumors using the BraTS dataset. This study also focused on privacy and applied different techniques to protect patient data [39]. Guo et al. proposed a cross-site modeling platform using FL for the reconstruction of MR images collected from multiple institutions using different scanners and acquisition protocols. The concealed features extracted from different sub-sites were aligned with the concealed features of the main site. The experiments were performed on multiple datasets with promising results [40]. Table 3 lists the effective methods used.

### 3.4. RQ2 Assessment: What Are the Metrics Used to Determine the Performance of Different Methods Applied to Brain Tumor Diagnosis?

The efficiency of a classifier is based on the sensitivity, specificity, precision, accuracy, and area under the curve (AUC). However, classification is not flawless, and images can be assigned to the wrong class. To test a classifier, the actual class of the image is required. The class assigned by the classifier is contrasted with the actual class to determine the classification accuracy [24]. The performance metrics are summarized in Table 4. 

#### 3.4.1. Performance Evaluation of Ensemble Methods

Sair et al. suggested a system that utilizes CNN with softmax. This study used a dataset of MRI images collected from 153 patients suffering from headaches, referred to the image center. The study included images of patients with brain tumors and normal patients. After examination by doctors, 1321 images were selected to validate the effectiveness of the proposed method [41]. The proposed method was tested on a single dataset, which lowers the credibility of the method. In comparison, Sultan et al. [42] presented a deep-learning-based method for brain tumor detection. The efficiency of their method was tested on two different datasets. The first dataset was acquired from brain tumor patients referred to the General Hospital from 2005 to 2010, and the second dataset was collected from Nanfang Hospital, Tianjin Medical University, China. The second dataset was acquired from the cancer imaging archive (TCIA) and the public repository of molecular brain neoplasia data (REMBRANDT). All the contrast-enhanced T1 weighted images added to the dataset were from 130 patients of different ages, races, diseases, and grades. This dataset has been publicly available since 2015. Many versions have been released by researchers. The latest version released in 2017 is commonly known as the Figshare dataset. The use of the two datasets enhanced the accuracy of the proposed method. Li et al. [43] proposed a novel method that combines information fusion with CNN. This study achieved a satisfactory result using the BraTS 2018 dataset consisting of 274 images of low-grade and high-grade tumors on MRI images of dimensions 155 × 240 × 240 pixels. A large image dataset was used to validate the proposed method. Noreen et al. [44] implemented an ensemble technique that incorporated Inception V3 with softmax and Dense-Net with softmax. To validate the effectiveness of the proposed method, a publicly available Figshare dataset was utilized. More publically available datasets must be used to validate the effectiveness of the proposed system. Liu et al. [45] used an encoder-decoder-based neural network technique, testing the efficiency of the model on the BraTS 2017 dataset. Additional publicly available datasets must be used to validate the accuracy of the proposed method. A process was suggested by Shakeel et al. [46] for effective image detection of brain tumors using machine-learning-based backpropagation neural networks (MLBPNN). This study used infrared technology for the detection of tumors less than 3 mm, undetectable in regular MRI images. Deng et al. [47] proposed a novel system that implements conditional random fields (CRF) with heterogeneous convolution neural networks (HCNN). This method achieved high accuracy in brain tumor detection on the BraTS 2013 dataset, which contains a limited number of brain tumor images. The validity of the proposed method must be established on multiple databases. In comparison to this, the combination of neutrosophy and convolutional neural networks proposed by Özyurt et al. [48] used TCIA to validate the proposed method. Multiple datasets must be used to check the effectiveness of the proposed method. Furthermore, Majib et al. proposed a novel idea that uses a visual geometry group (VGG) with a stack classifier. The effectiveness of the proposed method was tested on a privately collected dataset, achieving remarkable efficiency [49].The proposed method should be tested on one of the publicly available datasets. Amin et al. [50] proposed a statistical learning method that was tested on two publicly available datasets, BraTS 2013 and BraTS 2015, to demonstrate the strength of the proposed method. Mitta et al. [51] proposed a stationary wavelet transform (SWT) and a new growing convolution neural network (GCNN). To validate the proposed method, the cloud-based learning BRAINIX medical image database containing 2457 images was used. The dataset used in this study is only available on special request, the effectiveness of the proposed system must be tested on a publically available dataset. Saba et al. [52] proposed a grab cut method for the accurate segmentation of actual lesion symptoms. The transfer learning model VGG-19 was fine-tuned to acquire the features, which were subsequently concatenated with handcrafted (shape and texture) features through a serial-based method. The effectiveness of the proposed method was tested on three publicly available datasets: BraTS 2015, 2016, and 2017. Vijh et al. [37] implemented particle swarm optimization with CNN for the effective detection of brain tumors. For this purpose, the internet brain segmentation repository (IBSR) offers 61 sampling cases of T1-weighted brain magnetic resonance images to test the efficiency of proposed methods. In addition, 40 MS-free data sample images were taken from the Institute of Neurology and Genetics Nicosia, Cyprus, and the Laboratory of eHealth at the University of Cyprus. The images were normalized so that segmentation could be applied efficiently and properly. However, a publicly available dataset must be used to validate the performance of the proposed method. In comparison, Amin et al. [53] developed a process using a stack autoencoder in deep learning and tested the proposed method on publicly available datasets (BraTS 2012, 2013, 2014, and 2015). Rehman et al. [54] proposed a method that was based on a preprocessing step, a feed-forward pass CNN step, and a post-processing step, in which images were standardized and the field bias was fixed to remove false values across the skull region. This model was tested only on publicly available datasets from Figshare. Sharif et al. proposed a method in which Densenet 201 is fine-tuned by applying two different techniques: entropy–kurtosis-based high feature values (EKbHFV) and a modified genetic algorithm based on metaheuristics for feature selection. SVM cubic was used for classification. BraTS 2018 and BraTS 2019 were used to validate the proposed model [55]. 

#### 3.4.2. Performance Evaluation on Pretrained Method

Das et al. [56] proposed a novel CNN-based method that uses a publicly available dataset from Figshare containing 3064 T1-weighted contrast-enhanced images collected from 233 patients. The resolution of the images was 512 × 512 pixels. The data consisted of three different kinds of brain tumors: 708 meningiomas, 1426 gliomas, and 930 pituitary images. The main limitation is that only one dataset was used to measure the effectiveness of the proposed method. Gumaei et al. [57] proposed extreme learning for brain tumor diagnosis. This method achieved promising results when tested on a benchmark dataset of Figshare. Amin et al. [58] also proposed a novel deep neural network which was implemented on BraTS 2012, 2013, 2014, 2015, and ischemic stroke lesion segmentation (ISLES) 2015 and 2017 datasets. Toğaça et al. [59] proposed a brain MRNet method based on CNN. The database used in this analysis consisted of publicly available MR images labeled as normal and tumor. Field experts, such as doctors and radiologists, collected images from the dataset and shared them on the Internet. The total number of images was 253, and patients provided each image voluntarily, making the dataset heterogeneous. There were 155 malignant tumors and 98 normal tumor images. The images were not of a uniform resolution, and the image quality was not high, reducing the efficiency of the proposed method. Sajjad et al. [60] suggested a system that used CNN for brain tumor detection. In this study, the brain tumor dataset included 3064 contrast-enhanced T1 weighted images from 233 patients to test the accuracy of the proposed method. Multiple datasets must be used to test the accuracy of the proposed system. A novel idea was proposed by Nema et al. [61], who presented RescueNet for brain tumor segmentation and detection. The accuracy of the method was verified on BraTS 2015 and 2017 datasets. Sajid et al. [62] proposed a deep-learning-based method for tumor detection. The reliability of the method was checked on only the BraTS 2013 dataset. Multiple datasets must be used to validate the accuracy of the proposed method. Pernas et al. [63] proposed a deep-learning-based segmentation and classification method combined with a multiscale approach. Three spatial scales along with different processing pathways were used to process the input images. This method is based on the principle of the human visual system, and no preprocessing is required. The accuracy of the proposed method was validated on the Figshare dataset. The proposed system must be tested on a more publically available dataset.

#### 3.4.3. Performance Evaluation on Transfer Learning Method

Swati et al. [64] presented a method that combines content-based image retrieval (CBIR) with transfer learning (TL). For testing, the efficiency of the proposed method was demonstrated on a publicly available CE-MRI dataset. Adding more datasets will validate the performance of the proposed method. Sharif et al. [65] proposed a method that implements particle swarm optimization with feature fusion for brain tumor detection. BraTS datasets were utilized to validate the method. Amin et al. [66] suggested a process that transfers information using score-level fusion. The proposed study was tested on multiple BraTS 2012, 2013, 2014, and 2015 datasets. Sadad et al. [67] proposed a method that implements Resnet 50 with Unet architecture to perform effective segmentation. To improve the classification rate, data augmentation and preprocessing were introduced in this study, in addition to reinforcement learning via transfer learning for the multiple classifications of brain tumors. The accuracy of the proposed method was validated on the Figshare dataset.

#### 3.4.4. Performance Evaluation on Handcrafted Method

Teshnehlab et al. [68] implemented a fine-tuned CNN for brain tumor detection. This study used an image dataset collected from 200 patients, ranging in age from 6 to 80 years old. The total number of images in the dataset used to test the efficiency of the method was 1286. Sharif et al. [69] proposed an active deep neural network that was validated on four publicly available datasets, namely BraTS 2013, 2015, 2017, and 2018. These datasets contain high-grade glioma (HGG) and low-grade glioma (LGG) images. Deng et al. [70] implemented a CNN with non-quantifiable local texture for brain tumor detection. The reliability of the proposed method was tested on the BRATS 2015 dataset. The accuracy of a proposed method must be validated by using multiple datasets. A multi-channel 3D architecture map based on a deep neural network was proposed by Nje D et al. [71] to extract highly predicted features of tumors. Additional demographics were fed to a support vector machine to predict the final results. The proposed method used diffusion tensor imaging (DTI) and resting-state functional MRI (rs-fMRI) along with T1 enhanced images to compute multiple metric maps. This study used a limited number of images to test the accuracy of the proposed method. Publically available datasets must be used to validate the accuracy of the model. In addition, Sharif et al. [55] proposed a method that applied Densenet201 to train imbalanced data. In this method, the average pool layer extracts the features from the trained model for accurate classification; however, the properties of this layer are not sufficient. Therefore, for feature selection, the modified genetic algorithm (MGA) and EKbHFV were used. Subsequently, a non-redundant serial-based approach was used to fuse the features of both methods with SVM for classification. BraTS 2018 and BraTS 2019 were used to validate the efficiency of the proposed method.

### 3.5. RQ3 Assessment: What Types of Datasets Are Available to Diagnose Brain Tumors?

A wide range of data is available for the detection of brain tumors in selected studies. Some of them are publicly accessible, and some are available on special request.

#### 3.5.1. Benchmark Datasets

Benchmark datasets have been extensively utilized in selected studies for the diagnosis of brain tumors. Commonly used benchmark datasets are given below

#### 3.5.2. Figshare Dataset

This contains 3064 T1-weighted contrast-enhanced images collected from 233 patients with a resolution of 512 × 512 pixels. Data collected from three different kinds of brain tumors consisted of 708 meningiomas, 1426 gliomas, and 930 pituitaries images from the General Hospital and Nanfang Hospital, Tianjin Medical University, China [72].

#### 3.5.3. TCGA-GBM Dataset 

The cancer genome atlas glioblastoma multiforme (TCGA-GBM) consists of 500 different samples of brain cancer. It is an open-access dataset provided by the TCGA-GBM for researchers to conduct scientific studies on brain tumors. Therefore, this dataset does not require the approval of an ethics committee. In experimental studies, the images in the T1-weighted post-contrast (T1-gadolinium (Gd)) sequence were used to obtain the most realistic results in the tumor region of the brain MRI [73].

#### 3.5.4. BraTS 2012 & 2013 Datasets 

Both BraTS 2012 and 2013 have a total of 30 HGG/LGG patients’ data, which includes 20 HGG and 10 LGG cases [74,75].

#### 3.5.5. BraTS 2014 Dataset 

This dataset has data from 300 input subjects, of which 200 were used for the training of HGG cases and 100 were used for the testing of LGG cases [76].

#### 3.5.6. BraTS 2015 Dataset 

In BraTS 2015, there were a total of 284 subjects, of which 64 belonged to LGG and 220 belonged to HGG cases; of the total, 174 subjects were used for training and 110 subjects were used for testing [77].

#### 3.5.7. BraTS 2016 and 2017 Dataset 

The BraTS 2017 dataset consists of multimodal MRIs from various institutions. This dataset was designed to segment inherently heterogeneous brain tumors. The data are available for training, validation, and testing. The training package included 285 samples with manually annotated and confirmed ground values. Four modalities, including T1, T1c, T2, and FLAIR were provided for each sample, along with corresponding annotations; the image size was 240 × 240 × 155 pixels [78,79].

#### 3.5.8. BraTS 2018 Dataset 

BraTS 2018 also had 285 input subjects, of which 75 belonged to LGG and 210 belonged to HGG; 191 cases were used for testing. The dimensions of the images were 240 × 240 × 155 pixels [80].

#### 3.5.9. ISLES 2015 Dataset 

This dataset has 64 subjects with subacute stroke lesion segmentation (SISS). This dataset provided 42 subjects for training and 22 for testing [81].

#### 3.5.10. ISLES 2017 Dataset 

This dataset has 75 input subjects; 28 subjects were used for training and 36 for testing [82].

#### 3.5.11. Brain MRI Dataset 

This dataset has 253 input subjects that were collected from volunteer patients. The study included 155 tumor subjects and 98 normal subjects [83].

#### 3.5.12. BraTS 2019 Dataset 

Preoperative MRI scans are used in BraTS 2019, mainly focusing on gliomas (intrinsically complex brain tumors). The dataset also predicts the overall survival of patients [84].

### 3.6. Non-Public Dataset

Some datasets are not publicly available; they are mostly available on special request, as some researchers have built these datasets to investigate the efficacy of their proposed method. The following datasets are non-public datasets.

#### 3.6.1. Combined Dataset 

This dataset consists of 15,320 MRI images collected from different sources. The data are accessible upon request only under a license agreement [57].

#### 3.6.2. BRAINIX Dataset 

This data is only available on special request [85].

### 3.7. RQ4 Assessment: What Is the Quality of the Selected Papers?

In a systemic analysis, quality assurance has become an integral component. A questionnaire was formed which evaluates the quality of selected papers [18]. Two authors carried out the process of quality assessment for the selected studies.

(1) Has deep learning algorithms been used for diagnosing brain tumors? The response for the potential answer was ‘Yes (1)’ or ‘No (0)’.
(2) Does the research provide a simple approach for the detection of disease with data sets? The response for a potential answer was ‘Yes (1)’ or ‘No (0)’.
(3) A well-known and renowned publication source published the article. Quartile rankings (Q1, Q2, Q3, and Q4) were used to create the Journal Citation Reports, whereas (CORE) (A, B, and C) were used for computer science conference rankings

The potential answers to this question for conferences and seminars:

- 2.0 marks for CORE A-rank conference;
- 1.5 marks for CORE B rank conference;
- 1.0 mark for CORE C rank conference.

For journals, letters and scientific reports, the possible answers to this question were:

- 2.0 marks for Q1 rank journal;
- 1.5 marks for Q2 rank journal;
- 1.0 mark for Q3 rank journal;
- 0.5 marks for Q4 rank journal.

The quality criteria score (c) states that journals were considered more valuable than conferences and seminars, since the authors believe that publishing research in Q1, Q2, Q3, and Q4 could be more difficult as compared to other publications. The qualitative review of 55 selected studies has been presented in Appendix A.

### 3.8. RQ5 Assessment: What Is the Impact of the Selected Papers on Brain Tumor Detection?

Brain tumors have a similar appearance and structure, and due to this, the manual detection of brain tumors using MR images is very difficult and time consuming for the radiologist. The expert radiologist can easily differentiate between normal and abnormal MR images. However, the classification of these abnormal images into different tumor categories is still a challenging task [21]. A deep and federated-learning-based system can efficiently detect brain tumors and their type. Moreover, these studies help the radiologist to improve their diagnostic accuracy [40]. Brain tumors occur in irregular shapes, and it is a very difficult task for the radiologist to manually detect a specific type of tumor from MR images. Laukamp et al. [25] provided a model for the automated classification of grade I, grade II, and grade III meningiomas. Grade I is considered benign, whereas grade II and Grade III have a high risk of recurrence, invasiveness, and aggressiveness. In addition to this, Özyurt et al. [32] proposed a method that helps healthcare experts in the identification of benign and malignant tumors, whereas sultan et al. [42] proposed a method that enhances the efficiency of clinical experts by providing the multiclassification of brain tumors using MR images. Swati et al. [64] proposed a method that classifies the brain tumor into glioma, meningioma, and pituitary tumor with the minimum intervention of radiologists.

## 4. Discussion

**Figure 4:**
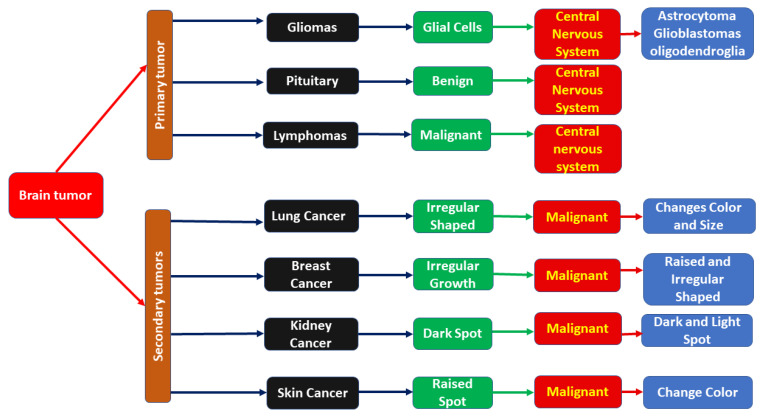
Taxonomy for brain tumor diagnosis.

**Figure 5:**
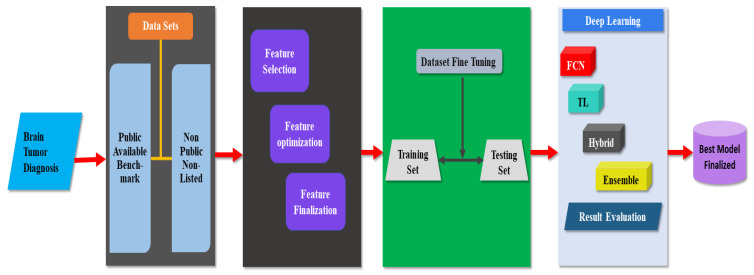
Common model for brain tumor diagnosis.

A comprehensive discussion is presented in this section, covering different methods of brain tumor diagnosis. To summarize the findings of this research, a taxonomy for brain tumor diagnosis is presented in Figure 4. A deep-learning-based brain tumor diagnosis model is also shown in Figure 5.

### 4.1. Taxonomy for Brain Tumor Diagnosis

A taxonomy was proposed by reviewing the selected literature. In the first step, brain tumors were classified into primary and secondary tumors. Primary tumors develop because of the abnormal growth of cells in the brain. These tumors are categorized as benign or malignant, depending on their type, and they can be life-threatening because when they grow, they cause damage to brain cells and skulls [14]. Primary tumors have many types, and they can develop from brain membranes, brain cells, nerve cells, and glands. Gliomas and meningiomas are the most common form of tumors [43] and originate from the cerebrum and supportive tissues of the brain [42,86]. The other types include pituitary tumors, pineal gland tumors, ependymomas, craniopharyngiomas, lymphomas, germ cell tumors, and schwannomas [87]. These tumors can cause vomiting, blurred vision, dizziness, memory loss, loss of balance, slow mental response, confusion, and difficulty in writing and reading [7]. Some of these tumors are cancerous, while others are non-cancerous. The majority of brain cancers consist of secondary brain tumors. Tumors initially develop in one part of the body and can spread to the brain. These tumors consist of lung cancer, breast cancer, kidney cancer, and skin cancer. Secondary tumors of the brain are malignant. Benign tumors are not distributed from one body part to another [1]. The taxonomy for brain tumor diagnosis is shown in Figure 4.

### 4.2. Common Model Used for Brain Tumor Diagnosis

The model was finalized after the analysis of best-performing methods existing in the literature, such as [43,52,53,58,66]. The strengths and weaknesses of each method were investigated. The model for the detection of brain tumors helps researchers overcome existing issues in current research, and it has been based on five major steps: dataset selection, analysis of the data for appropriate feature selection, identification of training and testing images, CNN-based model selection, and finalization of the best model. The initial phase is to obtain data from publicly accessible datasets and confidential data, including the images, gathered via the Internet for the diagnosis of brain tumors. The publicly available benchmark datasets, Figshare, BraTS challenge 2013, 2014, 2015, 2017, and 2018, ISLES 2015 and 2017, TCGA-GBM, and Brain MRI are accessible to all. In the non-public category, a combined dataset is available with images collected from different sources. Brainix is also available upon special request for brain tumor diagnosis. In addition, MRI image data available on the Internet have been utilized. Fine-tuning was applied to the images of different datasets, which eliminates irrelevant details that have been attached to the tumor area to achieve maximum performance. The proper selection of training and testing sets of images is of significant importance, and image testing and training sets were defined according to the proposed method. The appropriate selection of features is valuable because feature reduction technologies have been used to eliminate attributes to extract the most relevant ones from accessible data. Deep learning techniques were used to check the reliability of the techniques, following all the preceding steps. Several CNN-based techniques, such as ensemble methods, handcrafted methods, fully convolutional network methods, and hybrid methods are available, among which a suitable approach was selected to test the reliability of the proposed method. The common model is shown in Figure 5.

## 5. Open Issues and Challenges

The diagnosis of brain tumors using the CNN method is challenging. This segment deals with open issues and tasks that are discussed in the literature.

### 5.1. Dataset Variations

There are various available datasets for the diagnosis of brain tumors. Several datasets are publicly accessible, whereas others are not publicly accessible. Different image dimensions are used in different datasets [40].

### 5.2. Number of Images in Dataset

Every dataset has a varying number of low-grade and high-grade tumors. Some researchers created a dataset by collecting images from patients in different hospitals. These datasets vary in the number of images [55].

### 5.3. Size of Tumor

Tumor size is significant. It is difficult to identify the tumor if the size of the lesion is less than 3 mm. To address this challenge, Shakeel et al. [46] utilized infrared imaging technology to detect brain tumors of less than 3 mm.

### 5.4. Age of Patients

It was observed that in the literature, all the data are collected from patients with an average age between 40 and 70 years. However, no data were collected from younger patients [22].

## 6. Principal Findings

The principal findings were obtained after the analysis of the existing literature as provided below.

### 6.1. Best Classifiers for Brain Tumor Detection

It has been observed from the selected literature that ensemble and handcrafted classifiers show outstanding results for brain tumor detection.

### 6.2. Accuracy Evaluation of Classifiers

The training and test sets played a vital role in the accuracy of the classifier. It has been observed in the literature that the 75:25 ratio for training and testing provides the best accuracy [65].

### 6.3. Widely Used Datasets

It has been observed that in the literature, the BraTS challenge and Figshare (CE-MRI) datasets have been widely used by researchers.

## 7. Conclusions

In this study, a systematic literature review of brain tumor diagnosis was performed. A taxonomy was created to summarize the broad range of existing brain tumor diagnosis solutions. In addition, a common model was identified after analyzing existing studies, for researchers to better diagnose brain tumors. Open issues and challenges were also discussed to guide future researchers working in the field of brain tumor diagnosis. This study focused on deep learning and federated learning. Common techniques such as pre-trained models, fully convolutional neural networks (FCNs), handcrafted methods, and ensembles are widely used to diagnose brain tumors. With deep learning techniques, there is no dire need for composite and complex pre-processing techniques, such as image resizing, cropping, and normalization of the pixel values. Some studies have also used handcrafted features to perform pre-processing for the segmentation and extraction of features. Compared to conventional methods, deep-learning-based methods produce better results. Moreover, the issue of the limited dataset for training and testing is resolved by federated learning without compromising the privacy of data. One of the critical tasks in medical demographic images is proper labeling. A variety of benchmark datasets, such as the BraTS challenge 2013, 2014, 2015, 2017, 2018, and 2019 datasets, are available for researchers in this domain to validate their work on publicly available datasets. Researchers have also used non-public and self-collected datasets for the diagnosis of brain tumors. However, the diversity of available datasets makes it difficult to compare and validate the results. Furthermore, gender, age, and race were added to achieve better results. However, increasing the precision remains an obvious challenge. The primary goal is to significantly increase the sensitivity and improve the specificity of the methods and overall precision.

## Figures and Tables

**Table 1:** Research questions.

	Statement of Research Question	Motivation
Q1	What are the best available methods for the detection of a brain tumor?	This question investigates a deep and federated-learning-based method for the diagnosis of brain tumors.
Q2	What are the metrics used to determine the performance of different methods used for brain tumor diagnosis?	This question determines the research efficacy of deep learning and federated-learning-based methods for brain tumor diagnosis.
Q3	What datasets are used in recent research for the diagnosis of brain tumors?	This question identifies the available benchmark, public, and non-public datasets for brain tumor diagnosis.
Q4	What is the quality of the selected papers?	This question investigates the quality of the selected studies.
Q5	What is the impact of the selected papers on brain tumor detection?	This question investigates the impact of selected papers on the detection of brain tumors with a minimum intervention of radiologists

**Table 2:** Search strings for repositories.

Repository Name	Search Strings
ACM	((“deep learning” OR “machine learning” OR “artificial intelligence” OR “convolutional neural network” OR “federated learning”) AND (“glioblastoma,” OR “astrocytoma,” OR “brain cancer,” OR “brain tumor”) AND (“detection” OR “classification”)) Publication Year: 2017–2021
IEEE Xplore	((“document title”: “deep learning” OR “machine learning” OR “artificial intelligence” OR “convolutional neural network” OR “federated learning” OR “supervised learning” OR “Bayesian”) AND (“abstract”: “glioblastoma,” OR “astrocytoma,” OR “brain cancer,” OR “brain tumor”) AND (“detection” OR “classification”)) Publication Year: 2017–2021
Medline	(“deep learning”(All Fields) OR “machine learning”(All Fields) OR “artificial intelligence”(All Fields) OR “convolutional neural network”(All Fields) OR “federated learning”(All Fields) AND (“glioblastoma,”(All Fields) OR (“astrocytoma “(MeSH Terms) OR (“brain”(All Fields) AND “tumor”(All Fields)) OR “brain tumor”[All Fields] OR (“brain”(All Fields) AND “cancer”(All Fields)) OR “brain cancer”(All Fields)) OR “brain tumor”(All Fields)) AND (“detection”(All Fields) OR “diagnosis”(All Fields) OR “classification”(All Fields)) Publication Year: 2017–2021
Elsevier	(“deep learning” OR “machine learning” OR “artificial intelligence” OR “convolutional neural network” OR “federated learning”) AND (“glioblastoma,” OR “astrocytoma,” OR “brain cancer,” OR “brain tumor”) AND (“detection” OR “classification”) Publication Year: 2017–2021
Springer	((“deep learning” OR “machine learning” OR “artificial intelligence” OR “convolutional neural network” OR “federated learning”) AND (“glioblastoma,” OR “astrocytoma,” OR “brain cancer,” OR “brain tumor”) AND (“detection” OR “classification”)) Publication Year: 2017–2021
Scopus	TITLE-ABS-KEY (“deep learning” OR “machine learning” OR “artificial intelligence” OR “convolutional neural network” OR “federated learning”) AND (“glioblastoma,” OR “astrocytoma,” OR “brain cancer,” OR “brain tumor”) AND (“detection” OR “diagnosis” OR “classification”)) Year: 2017–2021
Wiley	deep-learning OR machine learning OR artificial intelligence OR convolutional neural network OR federated learning AND glioblastoma, OR astrocytoma, OR brain cancer OR brain tumor AND detection OR diagnosis OR classification Year: 2017–2021

**Table 3:** Methods for brain tumor diagnosis.

Publication Contribution	Architecture	Training Algorithm	Dataset	Source
A revamped CapsNet architecture for the detection of brain tumors that carry the coarse tumor borders into the extra pipeline to improve the emphasis of CapsNet.	CNN	CapsNets	CE-MRI	[23]
Automatic and efficient brain tumor segmentation and detection is achieved by using U-Net.	CNN	U-Net + Resnet50	BraTS 2015	[24]
The performance of the deep learning model was investigated on MRI data from various institutions.	CNN	Deep learning	BraTS benchmark	[25]
Two-stage cascaded U-Net for brain tumor detection and segmentation.	CNN	U-Net		[26]
Brain tumor classification by transferring CNN-based learning to SVM based classifier.	CNN	CNN + SVM	BraTS challenge 2019	[27]
Brain tumor classified using CNN, and for the segmentation of tumor, the watershed technique was implemented.	CNN	CNN + Watershed	Non-published brain MRI dataset	[28]
Fine and coarse features were extracted using hybrid two-path convolution with a modified down-sampling structure.	CNN	Hybrid two-path CNN	Non-published brain MRI dataset	[29]
CNN was used with the curvelet domain, which extracts features of reasonable resolution and direction.	CNN	CNN + Curvelet domain	BraTS 2017	[30]
Active contours used with CNN to automatically segment the tumor faster, independent of image type	DCNN	CNN + Active contour	Non-published brain MRI dataset	[31]
The fuzzy c-means efficiently segment the tumor, whereas pretrained SqueezNet effectively detects the tumor.	CNN	Fuzzy c-mean + SqueezNet	BraTS 2015	[32]
Accurate segmentation was achieved using triangular fuzzy median filters, whereas classification was done using ELM.	CNN	ST + ELM	BraTS 2012. 2013, 2014, 2015	[33]
Pre-processing, feature extraction, imaging classification and brain tumor segmentation were achieved using CNN with SVM.	CNN	CNN + SVM	40 MRI image dataset	[34]
Brain tumor detection using 3D semantic segmentation with conventional encoder–decoder architecture.	CNN	3D Semantic with encoder decoder	BraTS 2019	[35]
CNN and PNN have been utilized to make an intelligent system that can detect tumors of any shape and size efficiently.	CNN	PNN + CNN	BraTS 2013	[36]
Optimal threshold value with OTSU for the optimization of the adaptive swarm. This system uses an anisotropic diffusion filter to remove noise, while classification is done by the CNN.	CNN	CNN + OTSU	IBSR dataset + MS free brain dataset	[37]
Federated learning to improve the training process; for this purpose, multiple organizations collaborated with the privacy of patient data retained. The performance of federated semantic segmentation is demonstrated using a deep learning model.	FL	DNN	Different institutions; collaborated dataset	[38]
Federated learning based on deep neural network (DNN) for the segmentation of brain tumor using BraTS dataset.	FL	DNN	BraTS 2018	[39]
A cross-site modeling platform using FL for the reconstruction of MR images collected from multiple institutions using different scanners and acquisition protocols. The concealed features extracted from different sub-sites are aligned with the concealed features of the main site	FL	DNN	Multiple datasets	[40]

**Table 4:** Performance evaluation.

Classifier	Sensitivity /Recall	Specifity	Precision	Accuracy	Dice	Dataset	Source
CNN + SoftMax	100%	96.42%	98.83%	99.12%	-----	CE-MRI	[41]
CNN + GA	95.5%	98.7%	95.8%	97.54%	-----	Combined dataset	[42]
Information Fusion + CNN	99.81%	-----	92.7%	-----	92.7%	BraTS 2018	[43]
Inception V3 + SoftMax	-----	-----	99.0%	-----	99.34%	CE-MRI	[44]
Encoder-decoder neural network	-----	-----	-----	-----	89.28%	BraTS 2017	[45]
MLBPNN	95.10%	99.8%	-----	93.33%	-----	Infrared imaging technology	[46]
CRF—HCNN	97.8%	-----	96.5%	-----	-----	BraTS 2013 & 2015	[47]
NS—CNN	96.25%	95%	-----	95.62%	-----	TCGA-GBM dataset	[48]
VGG + Stack classifier	99.1%	-----	99.2%	-----	-----	Private collected	[49]
Statistical learning	92%	100%	-----	96%	96%	BraTS 2013	[50]
Statistical learning	91%	90%	-----	90%	95%	BraTS 2015	[50]
SWT + GCNN	98.23%	-----	98.81%	-----	-----	BRAINIX dataset	[51]
Handcrafted + Deep learning	99%	-----	-----	98.78%	96.36%	BraTS 2015	[52]
Handcrafted + Deep learning	100%	100%	100%	99..63%	99.62%	BraTS 2016	[52]
Handcrafted + Deep learning	-----	-----	-----	99.69%	95.06%	BraTS 2017	[52]
OTSU +CNN	-----	-----	-----	98%	-----	IBSR	[37]
Stack autoencoder in DL	88%	100%	-----	90%	94%	BraTS 2012	[53]
Stack autoencoder in DL	100%	100%		100%		BraTS 2012	[53]
Stack autoencoder in DL	100%	90%	-----	95%	100%	BraTS 2013	[53]
Stack autoencoder in DL	98%	96%	-----	97%	96%	BraTS 2014	[53]
Stack autoencoder in DL	93%	100%	-----	95%	98%	BraTS 2015	[53]
Ensemble	-----	-----	-----	98.69%	-----	CE-MRI	[54]
Densenet201 with EKbHFV & MGA	99.9%	-----	99.9%	99.9%	-----	BraTS 2019	[55]
CNN	94.56%	89%	93.33%	94.39%	-----	CE-MRI	[56]
Extreme learning	91.6%	-----	-----	-----	94.93%	CE-MRI	[57]
DNN	98.4%	98.4%	99.9%	98.6%	98.4%	BraTS 2012	[58]
DNN	99.8%	98.9%	98.9%	99.8%	99.8%	BraTS 2013	[58]
DNN	92.01%	95.5%	95.5%	93.1%	92.9%	BraTS 2014	[58]
DNN	95%	97.2%	-----	95.1%	96%	BraTS 2015	[58]
DNN	99.05%	98.20%	-----	100%		ISLES 2015	[58]
DNN	99.44%	100%	-----	98.8.7%	94.63%	ISLES 2017	[58]
Brain MRNet	96.0%	96.08%	92.31%	96.05%	84.2%	BrainMRI dataset	[59]
Pretrained CNN	88.41%	96.12%	-----	94.58%	-----	CE-MRI	[60]
RescueNet	94.89%	-----	-----	-----	94.29%	BraTS 2015	[61]
RescueNet	99%	-----	-----	-----	-----	BraTS 2017	[61]
Deep learning	90%	94%	-----	-----	88%	BraTS 2013	[62]
MultiScale CNN	94%			97.3%	82.8%	CE-MRI	[63]
CBIR—TL	-----	-----	96.13%	-----	-----	CE-MRI	[64]
Transfer learning	80%	98.1%	-----	97%	-----	BraTS 2015	[65]
Score Level Fusion using TL	95.31%	96.30%	-----	-----	96.44%	BraTS 2014	[66]
Score Level Fusion using TL	97.62%	95.05%	-----	-----	97.74%	BraTS 2013	[66]
Score Level Fusion using TL	-----	-----	-----	-----	-----	BraTS 2015	[66]
Score Level Fusion using TL	99.9%	-----	-----	-----	100%	BraTS 2016	[66]
Score Level Fusion using TL	91.27%	-----	-----	-----	99.80%	BraTS 2017	[66]
Resnet50 + Unet	-----	-----	-----	99.61%	-----	CE-MRI	[67]
Fine-tuned CNN)	94.64%	100%	-----	96.88%	-----	CE-MRI	[68]
Active DNN	-----	-----	-----	98.3%	-----	BraTS 2013	[69]
Active DNN	-----	-----	97.2%	97.8%	95.0%	BraTS 2015	[69]
Active DNN	98.39%	96.06%	-----	96.9%	99.59%	BraTS 2017	[69]
Active DNN	98.7%	99.0%	99%	92.5%	99.94%	BraTS 2018	[69]
CNN with non-quantifiable local texture	90.12%	-----	-----	-----	85.25%	BraTS 2015	[70]
Dtf + Fc7	88.9%	87.5%	-----	88%	-----	68% patient data collected from 2010–2015 Haushan hospital	[71]
Densenet with MGA +EKbHFV	99.7%	-----	99.7%	99.7%	-----	BraTS 2018	[55]
Densenet with MGA + EKbHFV	-----	-----	-----	99.8%	98.7%	BraTS 2019	[55]

## Data Availability

Not applicable.

## Appendix A

**Table A1:** Quality of selected papers.

Publication Number	Source of Publication	Publication Year	Criteria for Quality			
			a	b	c	Final Score
[23]	Conference paper	2019	0.5	1.0	1.5	3.0
[24]	Journal paper	2017	1.0	1.0	1.5	3.5
[25]	Journal paper	2019	1.0	1.0	1.5	3.5
[26]	Conference paper	2019	0.5	0.5	2.0	3.0
[27]	Conference paper	2019	0.5	0.5	2.0	3.0
[28]	Conference paper	2019	1.0	1.0	1.5	3.5
[29]	Conference paper	2019	1.0	1.0	1.5	3.5
[30]	Conference paper	2020	1.0	1.0	1.0	3.0
[31]	Conference paper	2017	1.0	1.0	1.5	3.5
[32]	Journal paper	2020	0.5	0.5	2.0	3.0
[33]	Journal paper	2020	1.0	1.0	2.0	4.0
[34]	Journal paper	2019	0.5	0.5	2.0	3.0
[35]	Conference paper	2019	1.0	1.0	1.5	3.5
[36]	Conference paper	2019	1.0	1.0	2.0	4.0
[37]	Journal paper	2020	1.0	1.0	1.5	3.5
[38]	Conference paper	2018	1.0	1.0	0.5	2.5
[39]	Conference paper	2019	0.5	0.5	1.5	2.5
[40]	Conference paper	2021	0.5	1.0	1.5	3.0
[41]	Conference paper	2019	1.0	1.0	2.0	4.0
[42]	Journal paper	2019	0.5	1.0	1.5	3.0
[43]	Journal paper	2019	1.0	0.5	2.0	3.5
[44]	Journal paper	2020	1.0	1.0	1.5	3.5
[45]	Journal paper	2020	0.5	1.0	1.5	3.0
[46]	Journal paper	2019	1.0	0.5	2.0	3.5
[47]	Journal paper	2020	1.0	1.0	0.0	2.0
[48]	Journal paper	2019	1.0	1.0	2.0	4.0
[49]	Journal paper	2021	1.0	1.0	2.0	4.0
[50]	Journal paper	2019	1.0	1.0	2.0	4.0
[51]	Journal paper	2019	1.0	1.0	2.0	4.0
[52]	Journal paper	2020	1.0	1.0	1.0	4.0
[37]	Journal paper	2020	1.0	1.0	2.0	4.0
[53]	Journal paper	2020	1.0	1.0	1.0	3.0
[54]	Journal paper	2020	1.0	1.0	1.0	3.0
[55]	Journal paper	2021	1.0	1.0	1.5	3.5
[56]	Conference paper	2019	1.0	1.0	1.0	3.0
[57]	Journal paper	2019	1.0	1.0	1.5	3.5
[58]	Journal paper	2018	1.0	1.0	1.5	3.5
[59]	Journal paper	2020	1.0	1.0	1.5	3.5
[60]	Journal paper	2019	1.0	1.0	1.5	3.5
[61]	Journal paper	2020	1.0	1.0	1.5	3.5
[62]	Journal paper	2019	1.0	1.0	2.0	4.0
[63]	Journal paper	2021	1.0	1.0	1.5	3.5
[64]	Journal paper	2019	1.0	1.0	1.5	3.5
[65]	Journal paper	2020	1.0	1.0	2.0	4.0
[66]	Journal paper	2019	1.0	1.0	2.0	4.0
[67]	Journal paper	2021	1.0	1.0	2.0	4.0
[68]	Conference paper	2019	1.0	1.0	2.0	4.0
[69]	Journal paper	2020	1.0	1.0	2.0	4.0
[70]	Journal paper	2019	1.0	1.0	2.0	4.0
[71]	Journal paper	2019	1.0	1.0	2.0	4.0
[55]	Journal paper	2021	1.0	1.0	2.0	4.0
